# Sociodemographic variations in the uptake of faecal immunochemical tests in primary care: a retrospective study

**DOI:** 10.3399/BJGP.2023.0033

**Published:** 2023-10-17

**Authors:** James A Bailey, Alastair J Morton, James Jones, Caroline J Chapman, Simon Oliver, Joanne R Morling, Heetan Patel, Ayan Banerjea, David J Humes

**Affiliations:** Nottingham Colorectal Service, Nottingham University Hospitals NHS Trust; School of Medicine, University of Nottingham, Nottingham.; Nottingham Colorectal Service, Nottingham University Hospitals NHS Trust; School of Medicine, University of Nottingham, Nottingham; National Institute for Health and Care Research (NIHR) Nottingham Biomedical Research Centre (BRC), Nottingham University Hospitals NHS Trust and the University of Nottingham, Nottingham.; Nottingham Colorectal Service, Nottingham University Hospitals NHS Trust, Nottingham.; Eastern Hub, Bowel Cancer Screening Programme, Nottingham University Hospitals NHS Trust, Nottingham.; Nottingham and Nottinghamshire Integrated Care Board, Nottingham.; NIHR Nottingham BRC, Nottingham University Hospitals NHS Trust and the University of Nottingham, Nottingham; School of Medicine, University of Nottingham, Nottingham.; Nottingham and Nottinghamshire Integrated Care Board, Nottingham.; Nottingham Colorectal Service, Nottingham University Hospitals NHS Trust, Nottingham.; Nottingham Colorectal Service, Nottingham University Hospitals NHS Trust; NIHR Nottingham BRC, Nottingham University Hospitals NHS Trust and the University of Nottingham, Nottingham.

**Keywords:** colorectal cancer, faecal immunochemical testing, inequality, social deprivation, primary health care, referral and consultation

## Abstract

**Background:**

Faecal immunochemical test (FIT) usage for symptomatic patients is increasing, but variations in use caused by sociodemographic factors are unknown. A clinical pathway for colorectal cancer (CRC) was introduced in primary care for symptomatic patients in November 2017. The pathway was commissioned to provide GPs with direct access to FITs.

**Aim:**

To identify whether sociodemographic factors affect FIT return in symptomatic patients.

**Design and setting:**

A retrospective study was undertaken in Nottingham, UK, following the introduction of FIT as triage tool in primary care. It was mandated for all colorectal referrals (except rectal bleeding or mass) to secondary care. FIT was used, alongside full blood count and ferritin, to stratify CRC risk.

**Method:**

All referrals from November 2017 to December 2021 were retrospectively reviewed. Sociodemographic factors affecting FIT return were analysed by multivariate logistic regression.

**Results:**

A total of 35 289 (90.7%) patients returned their index FIT, while 3631 (9.3%) did not. On multivariate analysis, males were less likely to return an FIT (odds ratio [OR] 1.11, 95% confidence interval [CI] = 1.03 to 1.19). Patients aged ≥65 years were more likely to return an FIT (OR 0.78 for non-return, 95% CI = 0.72 to 0.83). Unreturned FIT more than doubled in the most compared with the least deprived quintile (OR 2.20, 95% CI = 1.99 to 2.43). Patients from Asian (OR 1.82, 95% CI = 1.58 to 2.10), Black (OR 1.21, 95% CI = 0.98 to 1.49), and mixed or other ethnic groups (OR 1.29, 95% CI = 1.05 to 1.59) were more likely to not return an FIT compared with patients from a White ethnic group. A total of 599 (1.5%) CRCs were detected; 561 in those who returned a first FIT request.

**Conclusion:**

FIT return in those suspected of having CRC varied by sex, age, ethnic group, and socioeconomic deprivation. Strategies to mitigate effects on FIT return and CRC detection should be considered as FIT usage expands.

## INTRODUCTION

Colorectal cancer (CRC) is common, with over 42 000 new cases and 16 000 deaths in the UK annually.^1^ Survival is related to stage,^2^ with 90% of early stage diagnoses surviving >5 years compared with 10% diagnosed at advanced stage.^3^ Population- based screening of asymptomatic patients and expedited diagnostic pathways for patients with symptoms aim to improve outcomes. Screening is cost-effective, reducing CRC mortality^4^^,^^5^ by diagnosing earlier- stage disease, but most diagnoses follow symptomatic referrals where similar improvements have not been achieved.^6^^,^^7^

The faecal immunochemical test (FIT) is used in the Bowel Cancer Screening Programme (BCSP), detecting occult faecal blood that indicates increased risk of CRC. More recently, FITs have been evaluated in patients with lower gastrointestinal symptoms following National Institute for Health and Care Excellence (NICE) guidance,^8^ identifying patients with the highest CRC risk for expedited investigation.^9^^–^^17^

A clinical pathway for CRC was introduced in 2017 in four local clinical commissioning groups (CCGs) in Nottingham to give GPs direct access to FIT. Its introduction increased the proportion diagnosed on CRC 2-week- wait (2WW) pathways.^18^ Early outcomes reported at that time suggested a higher proportion of patients diagnosed at an earlier stage; however, low numbers of patients included in that study, and the confounding effects of the COVID-19 pandemic, merit further study into any stage shift achieved by FITs, which is ongoing. New guidelines recommend urgent referral for those with an FIT result >10 µgHb/g faeces.^19^ Clinicians are advised for those below this level to consider alternate cancer diagnoses, routine referral, or safety netting in primary care. Higher FIT return rates have been reported in symptomatic populations;^15^^,^^16^^,^^20^ however, sociodemographic variations in symptomatic FIT uptake are a research priority.^19^

**Table:** How this fits in

Faecal immunochemical tests (FITs) are increasingly used to triage patients with symptoms suggestive of colorectal cancer but variations in use by demographics, ethnic group, and socioeconomic status are unknown. This study’s large regional dataset has shown that male patients, those aged <65 years, the most deprived patients, and ethnic minority groups are less likely to return an FIT sample. It is important that strategies are developed to ensure patients with these protected characteristics are not disadvantaged with the increasing usage of FIT to prioritise urgency of investigations.

There is considerable sex-based, ethnic, and socioeconomic variability in CRC diagnosis and treatment.^21^^,^^22^ Differential screening participation rates are related to sociodemographic factors.^22^^–^^25^ Screening participation varies by ethnic group, suggesting complex interactions between socioeconomic, cultural, and physician factors.^26^^,^^27^ Participation is lower for males, those living in deprived areas, and certain ethnic groups,^26^^,^^28^^–^^30^ whereas CRC is more common in males and deprived groups.^1^ CRC incidence is lower in Asian and Black populations,^31^ but outcomes are worse.^26^

These differences in screening participation have not been demonstrated in symptomatic populations. Patient concern may explain higher returns in symptomatic pathways (∼90%)^20^ than screening (∼65%).^21^^,^^32^ Understanding sociodemographic factors in uptake is important when patients from ethnic minority and deprived backgrounds have poorer outcomes,^5^^,^^26^ especially as FIT usage in symptomatic pathways increases.^8^^,^^19^ The study aimed to evaluate whether sociodemographic factors affect FIT return in symptomatic pathways.

## METHOD

### Study population

FIT was introduced as a triage tool for all adult symptomatic patients in 2017 (excluding rectal bleeding or mass).^20^^,^^33^ The pathway was commissioned to provide direct access to FIT for GPs, requesting and acting on results independently or submitting a secondary care referral (including mandatory FIT and blood results). All FIT requests for patients with symptoms were recorded prospectively from pathway inception on 3 November 2017 to 31 December 2021. FIT return was reviewed retrospectively. FIT return was defined as returning a sample after the first request. FIT non-return was defined as no return by 14 days after the request. GPs were informed electronically if samples were not returned, recommending a further FIT request. Subsequent FIT requests made for first-test non-returners were analysed as a subgroup.

FIT requests were submitted via an electronic request system (ICE), with guidance provided on interpretation. FIT kits were sent or returned via post and analysed in a BCSP-accredited laboratory. The OC-Sensor FIT system was used to analyse all samples (see Supplementary Information S1).

### Exposures, covariates, and outcomes

A 65-year threshold was used to assess return between age groups, owing to the categorisation used in primary care datasets locally. Sex was classified as female, male, or unknown. Patient ethnic group was recorded as declared by the patient on the Patient Administration System (see Supplementary Information S2). Ethnic groups were categorised into the following five broad groups (defined by the UK Government for census research purposes): White; Asian or Asian British; Black, African, Caribbean, or Black British; mixed/multiple or other ethnic groups; and unknown. Socioeconomic data were obtained from six-digit postcodes using the Index of Deprivation tool (IoD2019) to derive Index of Multiple Deprivation (IMD) quintiles, from least (5th quintile) to most deprived (1st quintile). Base population data were obtained from NHS Nottingham and Nottinghamshire CCG. Patients with missing data were categorised as ‘unknown’. The primary outcome was FIT return or non-return. Cancer Outcomes and Services Dataset was used to evaluate the diagnosis of colorectal cancer: ICD codes C18–C20 (excluding C18.1).

### Thresholds

**Figure 1.**
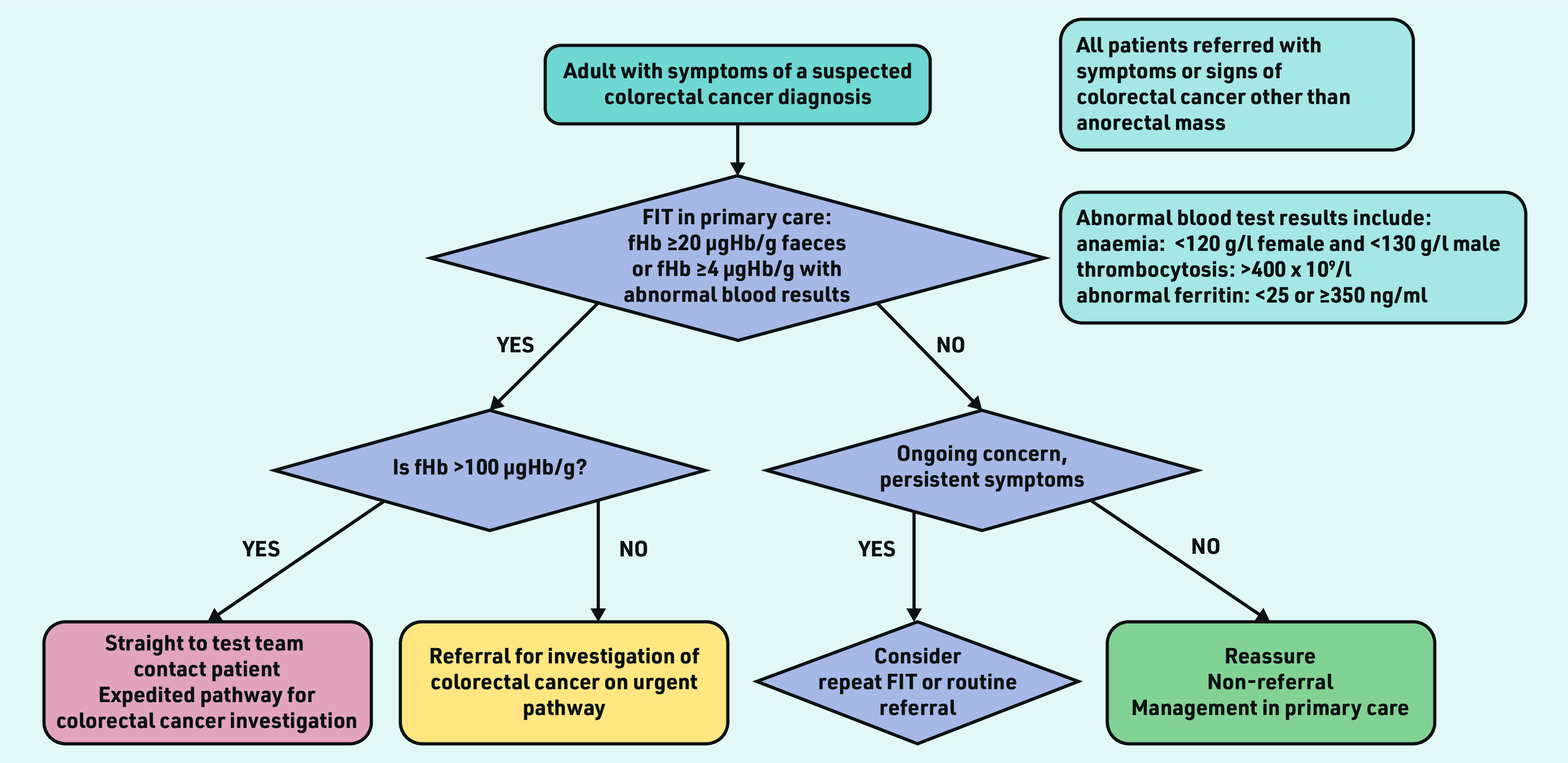
*Clinical pathway in Nottingham 2020–2022. fHb = faecal haemoglobin. FIT = faecal immunochemical test.*

The threshold for urgent investigation in patients with anaemia, abnormal ferritin, or thrombocytosis was ≥4 µgHb/g faeces. In March 2020, the threshold for urgent investigation for patients with normal haemoglobin, ferritin, and platelet count increased from 10 to 20 µgHb/g faeces. The clinical pathway for 2020–2022 is shown in Figure 1.

### Statistical analysis

Demographics were presented as proportions, stratified by FIT return. Histograms were constructed to assess distribution for continuous data. Means were calculated for parametric and medians for non-parametric data. Differences in proportions between groups were evaluated using χ^2^. Study population characteristics were compared with Nottinghamshire population data using χ^2^.

Factors predicting FIT non-return were evaluated using χ^2^. Univariate then multivariate logistic regression analyses were undertaken to evaluate FIT return or non-return by sex, age, ethnic group, and socioeconomic deprivation, adjusted for other significant variables. Age was treated as a categorical variable (18– 64 years and ≥65 years). CRC outcomes were examined first by χ^2^ comparison, and subsequently analysed within a univariate and multivariate model to report the CRC probability in FIT non-returners compared with the overall referred population and those returning a ‘negative’ FIT.

Stata (version 17) was used for analysis, with significance if *P*<0.05.

## RESULTS

### Cohort demographics

**Figure 2.**
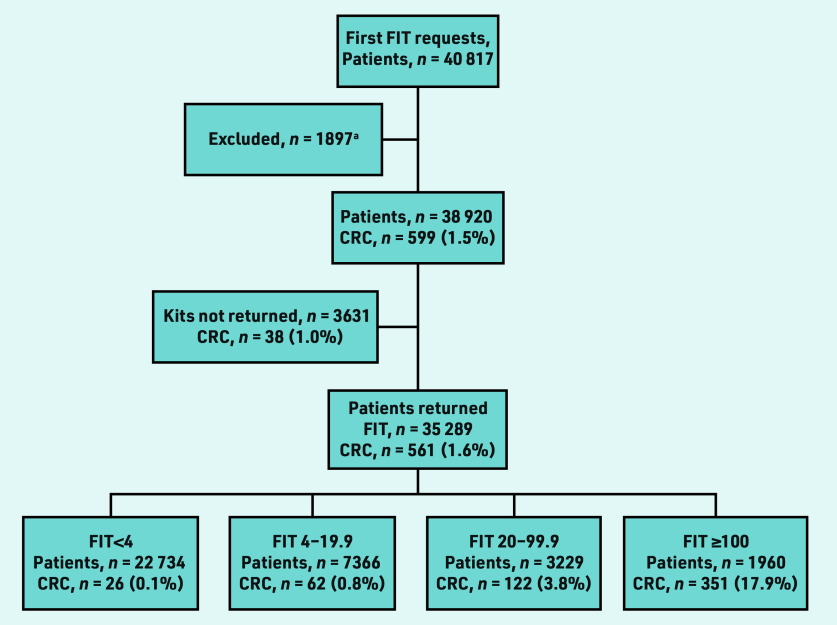
*Flowchart showing first faecal immunochemical test (FIT) requests made per patient, returns, and colorectal cancer diagnoses by FIT strata. ^a^ See Table 1 for reasons.*

A total of 49 166 FITs were requested for 40 817 individual patients in the study period (Figure 2). There were 1897 ineligible requests, which were excluded (Table 1). The first FIT requests for 38 920 individual patients were included in the main analysis. A total of 35 289 patients returned an FIT sample after the first request (90.7%). Of the 3631 non-returners, 1637 (45.1%) had a subsequent request within 6 months (data not shown). After a second request, 1022 of these patients (62.4%) returned an FIT sample. Twenty CRCs were detected in 1826 patients (1.1%) who had no further FIT requests made, despite an alert being made to GPs of non- return. Median follow up was 17.9 months (interquartile range [IQR] 8.8– 30.4); 14.2 months for non- returners (IQR 6.2–26.6) and 19.0 months for those with an fHb <4 µgHb/g faeces (IQR 9.6– 31.9). The median age was 66 years (IQR 54–77). The largest ethnic group was White (*n* = 27 277, 70.1%) (Table 2). The largest socioeconomic group of the investigated population was the least deprived quintile (*n* = 11 036, 28.4%).

**Table 1.** Excluded FIT requests

**Reason for exclusion**	**Excluded, *n***
Rectal bleeding	1218
Duplicate request	315
Request from out of area	197
Sampling error	101
Incomplete request	39
Not indicated, aged <18 years	16
Incomplete records	11
Total excluded	1897

*FIT = faecal immunochemical test.*

**Table 2.** Baseline characteristics of patients who had an FIT request from November 2017 to December 2021 compared with baseline Nottinghamshire population

	**Base population, *n* (%)**	**Investigated population, *n* (%)**	

**Category**	**Total**	**Total**	**CRC detected**
**Sex**			
Female	496 525 (49.9)	21 800 (56.0)	252 (1.2)
Male	498 755 (50.1)	17 112 (44.0)	347 (2.0)
Unknown	35 (0.004)	8 (0.02)	0 (0.0)

**Age, years**			
<65	777 085 (78.1)	18 029 (46.3)	130 (0.7)
≥65	218 195 (21.9)	20 891 (53.7)	469 (2.2)
Unknown	35 (0.004)	0 (0.0)	0 (0.0)

**Ethnic group**			
White	753 845 (75.7)	27 277 (70.1)	439 (1.6)
Asian	66 220 (6.7)	1584 (4.1)	6 (0.4)
Black	29 565 (3.0)	801 (2.1)	7 (0.9)
Mixed or other	31 750 (3.2)	876 (2.3)	8 (0.9)
Unknown	113 935 (11.4)	8382 (21.5)	139 (1.7)

**IMD quintile**			
5 (least deprived)	195 680 (19.7)	11 036 (28.4)	183 (1.7)
4	204 595 (20.6)	6278 (16.1)	124 (2.0)
3	205 315 (20.6)	6454 (16.6)	95 (1.5)
2	194 310 (19.5)	6177 (15.9)	95 (1.5)
1 (most deprived)	195 325 (19.6)	8927 (22.9)	102 (1.1)
Unknown	90 (0.01)	48 (0.1)	0 (0.0)

*FIT = faecal immunochemical test. IMD = Index of Multiple Deprivation.*

### Comparison with the Nottinghamshire population

There were significantly more females in the study compared with the Nottinghamshire population (56.0% versus 49.9%, *P*<0.001). The study population was older; 53.7% aged ≥65 years compared with 21.9% of the base population (*P*<0.001). There were differences between the ethnicities of the study and Nottinghamshire populations (*P*<0.001), the largest of which was in the unknown group (21.5% of the study population, 11.4% of Nottinghamshire). Social deprivation differed significantly (*P*<0.001). The least deprived (5th quintile) were over-represented in the study population, accounting for 28.4% of all FIT requests while constituting just 19.7% of the Nottinghamshire population. The most deprived quintile accounted for 22.9% of all FIT requests and represented 19.6% of the Nottinghamshire population (Table 2).

### FIT return

FIT return varied by sex, age, ethnic group, and social deprivation (Table 3). Males had lower return; 90.2% compared with 91.0% in females (*P* = 0.01). Non- returners were younger (median aged 62 years, IQR 49–77) than FIT returners (median age 67 years, IQR 55–77) (data not shown). FIT return in patients aged <65 years was lower than in those aged ≥65 years (89.2% versus 91.9%, *P*<0.001) (Table 3). FIT return was significantly higher for the White ethnic group (91.2%) compared with ethnic minority groups (83.8% for Asian patients, 86.6% for Black patients, and 87.2% for patients from mixed or other races, *P*<0.001). FIT return was lower in the most deprived quintile (86.3%) compared with the least (93.6%, *P*<0.001).

**Table 3.** Univariate and multivariate logistic regression analysis of FIT return by sex, age, ethnic group, and social deprivation

**Category**	**Return, *n* (%)**	**Non-return, *n* (%)**	**Univariate OR (95% CI)**	**Multivariate OR (95% CI)**
**Sex^a^**				
Female	19 841 (91.0)	1959 (9.0)	Reference	Reference
Male	15 442 (90.2)	1670 (9.8)	1.10 (1.02 to 1.17)	1.11 (1.03 to 1.19)

**Age, years**				
<65	16 080 (89.2)	1949 (10.8)	Reference	Reference
≥65	19 209 (91.9)	1682 (8.1)	0.72 (0.67 to 0.77)	0.78 (0.72 to 0.83)

**Ethnic group**				
White	24 864 (91.2)	2413 (8.8)	Reference	Reference
Asian	1328 (83.8)	256 (16.2)	1.99 (1.73 to 2.29)	1.82 (1.58 to 2.10)
Black	694 (86.6)	107 (13.4)	1.59 (1.29 to 1.96)	1.21 (0.98 to 1.49)
Mixed or other	764 (87.2)	112 (12.8)	1.51 (1.23 to 1.85)	1.29 (1.05 to 1.59)
Unknown	7639 (91.1)	743 (8.9)	1.00 (0.92 to 1.09)	0.99 (0.90 to 1.08)

**IMD quintile**				
5 (least deprived)	10 328 (93.6)	708 (6.4)	Reference	Reference
4	5808 (92.5)	470 (7.5)	1.18 (1.05 to 1.33)	1.18 (1.04 to 1.33)
3	5885 (91.2)	569 (8.8)	1.41 (1.26 to 1.58)	1.39 (1.24 to 1.56)
2	5521 (89.4)	656 (10.6)	1.73 (1.55 to 1.94)	1.68 (1.50 to 1.87)
1 (most deprived)	7703 (86.3)	1224 (13.7)	2.32 (2.10 to 2.55)	2.20 (1.99 to 2.43)
Unknown	44 (91.7)	4 (8.3)	1.30 (0.47 to 3.62)	1.28 (0.46 to 3.57)

a *Eight requests for patients of unknown sex with six samples returned not displayed in Table. FIT = faecal immunochemical test. IMD = Index of Multiple Deprivation.*

### Predictors of FIT return

Male patients were less likely than female patients to return an FIT, after adjustment for other factors (OR 1.11 for non-return, 95% CI = 1.03 to 1.19). Patients aged ≥65 years were more likely to return an FIT compared with those aged 18–64 years (OR 0.78, 95% CI = 0.72 to 0.83 for non- return). People from Asian and Black ethnic groups had a 1.8- and 1.2-fold increased FIT non- return rate compared with people from a White ethnic group (OR 1.82, 95% CI = 1.58 to 2.10 and OR 1.21, 95% CI = 0.98 to 1.49, respectively). FIT non-return was higher in the mixed or other ethnic group (OR 1.29, 95% CI = 1.05 to 1.59) but not the unknown ethnic group (OR 0.99, 95% CI = 0.90 to 1.08), compared with the White ethnic group. FIT non- return increased across each increasing deprivation quintile. After adjustment for confounders, the most deprived quintile were more than twice as likely to not return an FIT than the least deprived quintile (OR 2.20, 95% CI = 1.99 to 2.43) (Table 3).

### CRC diagnosis

A total of 599 CRCs were detected in the overall study population (1.5%), 561 in FIT returners (1.6%) and 38 (1.0%) in 3631 first FIT non-returners (Figure 2). In FIT non- returners, 20 CRCs were detected from 1826 patients via routine or emergency pathways after no further FIT requests were made (data not shown). Eighteen were detected in 1805 patients who had a further FIT requested (16 of these from 1637 patients having re-request within 6 months of initial request).

Non-returners after first FIT request were significantly more likely to be diagnosed with CRC than patients returning an FIT <4 (1.0% versus 0.1%, *P*<0.001) or FIT <20 (1.0% versus 0.3%, *P*<0.001) (Figure 2).

Patients who returned their first FIT request were significantly more likely to be diagnosed with CRC than patients returning an FIT after a further request was made (1.6% versus 1.0%, *P* = 0.05) (Figure 2). Patients who did not return their first request were significantly more likely to have a delay in diagnosis than patients returning their first request (*P* = 0.024; Supplementary Table S1).

## DISCUSSION

### Summary

To the authors’ knowledge, this is the first study to describe sociodemographic variations in FIT return in symptomatic patients from primary care. The study identified clear demographic, ethnic, and socioeconomic variations in FIT return, and clinicians need to be aware of these when requesting FITs, counselling patients, and safety netting in practice. Fewer male patients had an FIT requested compared with female patients, and males were less likely to return an FIT than females. Return was lower in younger patients (aged <65 years) and ethnic minority groups. The least deprived patients were over- represented in the referred population. FIT return decreased with increasing deprivation.

### Strengths and limitations

The large cohort is a strength of this study. The data presented are from primary care, representing an unselected real- life experience of FIT usage in patients consulting with symptoms.

One limitation included the large proportion of unknown ethnicity in the referred population, limiting further comparisons of outcomes with the base population. The proportions of known ethnicities are shown in Supplementary Table S2. FIT was not used locally for rectal bleeding or rectal mass in this period and cancer diagnoses in other trusts would not be captured, but this number is expected to be small. The first FIT request for each patient was considered to yield accurate cohort risks: subgroup analysis of additional requests did not identify divergence in return rate or test performance. Over-representation of patients living in least deprived areas in the referred population is in line with screening studies, with lowest engagement in the most deprived.^23^^,^^24^ This may be owing to patients from deprived areas presenting less to primary care or being less likely to be referred by GPs. Symptomatic patients may be more motivated to complete an FIT than asymptomatic patients owing to a perceived threat to their health. This may overcome negative emotions associated with lower engagement such as embarrassment, disgust, and fear.^34^^,^^35^ This reinforces the need to counsel patients when requesting FITs, promoting a more positive view of cancer outcomes to minimise fear-related avoidance.

### Comparison with existing literature

The lower referrals and FIT return rate for males represents a well-described trend of lower male engagement with healthcare services. Numerous explanations exist for this trend, including masculinity ideologies,^36^ fearful health beliefs, and lower health awareness.^37^ Practical systems-based solutions, such as pro- active follow up of patients after non- return, may yield higher engagement than strategies targeting the patient to change behaviour.^38^^,^^39^ Solving this imbalance may meaningfully reduce CRC mortality, given higher incidence and more pronounced screening disparities for males.^32^

Patients aged <65 years were less likely to return an FIT. This has reinforced the need to engage younger patients in whom CRC incidence is rising.^40^^,^^41^ Thorough counselling of risk at the time of FIT request is imperative when used in younger individuals, especially those who may rightly assume their absolute risk of CRC is lower until a high FIT result modifies that risk. This group face delayed diagnosis if FIT return is not actively encouraged. FITs represent an opportunity to identify high- risk younger patients, reducing missed curable pathology for those whom early stage diagnosis has the largest survival benefit.

FIT return was highest in patients from a White ethnic group and lowest in ethnic minorities. Ethnic minority groups and non- English speakers appear less likely to return FIT, as demonstrated in screening.^22^^,^^24^ CRC is less common in patients from Asian and Black ethnic groups in the UK,^31^ but often presents at a later stage.^26^ This disparity demands novel strategies to minimise ethnic inequalities, with appropriate safety netting and counselling.^42^ Recently, visual instructions have been introduced in multiple languages to address this barrier to healthcare participation in linguistically diverse populations. Further work on other communication challenges, such as difficulties with hearing or vision, is required.^43^^,^^44^ Focused media campaigns, including social media, may have a role, but surveyed preference for FIT is lower in patients who are younger (aged <65 years) and from non- White ethnic backgrounds.^45^

### Implications for practice

There is understandable interest in the CRC risk for patients who are ‘FIT- negative’ in primary care. The rate of CRC for FIT non- returners (1.0%) is lower than the 3% threshold defined by NICE^8^ for urgent referral, but far higher than those who are ‘FIT-negative’ with an fHb <4 µgHb/g (0.1%), as shown in Figure 2. Patients who returned an FIT after a further request was made had a similarly lower rate of CRC (1.0%) compared with those returning their first request (1.6%). Awareness in primary care of groups less likely to respond may reduce missed diagnoses more effectively than current concerns around patients who are ‘FIT-negative’. Frank conversations around willingness to sample faeces in at-risk groups and additional safety- netting strategies are advisable. Access to secondary care investigation for non-returners should underpin FIT implementation in primary care. Reported CRC rates in this subgroup suggest routine referral may be an appropriate safety net for FIT non-return, provided there is a robust system in place to alert GPs to FIT non-return and mitigate any risk to patients where the index of suspicion for CRC is high.

In conclusion, FIT usage in primary care appears to be broadly acceptable to patients with >90% return. FIT non-return is related to sex, age, ethnic group, and socioeconomic deprivation, with similar patterns to screening programmes. Disparities should be considered as FITs for symptomatic patients continue to expand, ensuring patients with these protected characteristics are not disadvantaged.

## Data

Data used in this article belong to Nottingham University Hospitals NHS Trust and cannot be provided by the authors.
